# Spatiotemporal dynamics and genotypic shift of Coxsackievirus A16 in Fujian, China, from 2010 to 2025

**DOI:** 10.3389/fmicb.2026.1893929

**Published:** 2026-08-11

**Authors:** Ying Zhu, Wenxiang He, Linfeng Li, Shaoyong Xu, Shujing Li, Mezige Qiesha, Jianfeng Xie, Wei Chen

**Affiliations:** 1 Fujian Provincial Center for Disease Control and Prevention, Fuzhou, Fujian, China; 2 Fujian Provincial Key Laboratory of Zoonosis Research, Fuzhou, Fujian, China; 3 School of Public Health, Fujian Medical University, Fuzhou, Fujian, China

**Keywords:** B1c, coxsackievirus A16, epidemiology, genetic evolution, hand, foot and mouth disease

## Abstract

**Introduction:**

Despite the diverse etiology of hand, foot, and mouth disease (HFMD), Coxsackievirus A16 (CVA16) persists as a dominant pathogen in the Asia-Pacific region. This study characterizes the molecular epidemiological evolution of CVA16 in Fujian Province from 2010 to 2025 to understand its changing prevalence and genetic evolution.

**Methods:**

We analyzed the temporal, spatial, and demographic distributions of CVA16-HFMD in Fujian Province from 2010 to 2025, and further investigated the phylogenetic characteristics via MEGA and BEAST software.

**Results:**

From 2010 to 2025, laboratory-confirmed CVA16 cases accounted for 25.96% of HFMD pathogen spectrum in Fujian, with periodic biennial fluctuations characterized by increases in even-numbered years and decreases in odd-numbered years and a post-2023 surged to >50%. CVA16 Cases peaked during spring–summer, with a male-to-female ratio of 1.69:1, predominantly in children aged 1–3 years. Genetically, B1 subgenotype (cluster B1a-c) co-circulated. B1b dominated before 2018, gradually shifting to B1a thereafter, and to B1c (emerging in late 2023) after mid-2023.

**Conclusion:**

These findings clarify the long-term distribution and genetic shifts of CVA16 in Fujian, identifying the emerging B1c as a major driver of recent outbreaks and providing a baseline for regional HFMD prevention and vaccine development.

## 1 Introduction

Hand, foot, and mouth disease (HFMD) is a common pediatric syndrome caused by enteroviruses, predominantly affecting children under 5 years of age. While typically presenting as a self-limiting illness, HFMD can progress to severe systemic complications, including brainstem encephalitis, meningoencephalitis, and acute flaccid paralysis. The primary pathogens include enterovirus A71 (EV-A71), coxsackievirus A16 (CVA16), coxsackievirus A6 (CVA6), and coxsackievirus A10 (CVA10) (Ji et al., 2019; Zhu et al., 2023; Mo et al., 2026). In routine surveillance, genotypes other than EV-A71 and CVA16 (including CVA6, CVA10, CVA4, etc.) are traditionally grouped as “other enteroviruses” (other EVs). Since 1997, EV-A71 and CVA16 have been responsible for several large-scale outbreaks in the Asia-Pacific region. However, recent studies have indicated that CVA6 and CVA10 are gradually replacing them as the predominant pathogens (Huang et al., 2024). CVA16 was first identified in Toronto, Ontario, Canada (Robinson et al., 1958), and has been continuously circulating in Asia since 2000 (Ganorkar et al., 2017; Chen et al., 2021; Nhu et al., 2021; Noisumdaeng and Puthavathana, 2023; Zhu et al., 2023; Huang et al., 2024). Notably, since 2016, CVA16 has experienced a resurgence in mainland China (Liu et al., 2025), yet, underlying drivers of this renewed prevalence remain elusive.

CVA16 belongs to the genus *Enterovirus* within the family *Picornaviridae*. Based on the VP1 gene, it is classified into three genotypes: A, B, and D (Han et al., 2020, 2023). Genotype B is further subdivided into two subgenotypes, B1 and B2, with the B1 comprising three clusters: B1a, B1b, and B1c. While genotypes A, D, and B2 are only sporadically detected, the B1 subgenotype is globally distributed (Han et al., 2023; Zhu et al., 2023). Beyond its broad geographical reach, CVA16 poses a substantial public health threat. Recent studies shown that newly emerged clusters (e.g., B1c) possess the potential to trigger regional outbreaks and achieve dominance (Zeng et al., 2025). Furthermore, surveillance data showed a rising positive detection rate of CVA16 in severe cases (Liu et al., 2025). Consequently, CVA16 remains a critical priority for public health research.

Within this global epidemiological context, China has served as one of the primary seeding populations for the global transmission of CVA16 (Han et al., 2023). Domestically, Eastern China played a decisive role in seeding the diffusion of CVA16 nationwide, with significant spatiotemporal clustering of epidemics (Han et al., 2020). Located along the southeastern coast, Fujian Province lies within this key transmission area where CVA16 epidemic activity has increased markedly in recent years. However, long-term epidemiological and phylogenetic data in Fujian remain limited.

This study aims to characterize the epidemiological and phylogenetic features of CVA16-associated HFMD in Fujian Province from 2010 to 2025. By elucidating the shifting epidemic trends and viral evolutionary patterns, our findings seek to provide a scientific foundation for the surveillance, prevention, and control of HFMD, as well as the strategic development of targeted vaccines.

## 2 Materials and methods

### 2.1 Ethics statement

The study was reviewed by the Ethical Review Committee of the Fujian Center for Disease Control and prevention (CDC) and determined to be exempt from formal ethical approval. The studies were conducted in accordance with the local legislation and institutional requirements. Specimens were collected and submitted by municipal CDCs in accordance with a standardized protocol provided by Chinese Center for Disease Control and Prevention. Given that the specimens were derived from routine clinical examinations at sentinel hospitals, the requirement for written informed consent was waived. All patient data were anonymized prior to analysis to ensure strict confidentiality.

### 2.2 Samples collection

In accordance with the Guidelines for Prevention and Control of Hand, Foot, and Mouth Disease (2009 Edition) (Chinese Center for Disease Control and Prevention, 2009), we analyzed HFMD cases in Fujian Province occurring between January 1, 2010 and December 31, 2025 with complete data on epidemiological characteristics including sex, age, onset time and geographic location. All epidemiological information and clinical specimens—processed for nucleic acid testing and viral isolation per the guidelines—were routinely collected and submitted by municipal CDCs. Descriptive statistical analyses of the data were performed using SPSS 26.0.

### 2.3 Sequencing and molecular typing

The VP1 region of clinical samples was amplified as previously described (Chen et al., 2020), while that of isolates was amplified using primers CVA16-VP1-F/R (Chinese Center for Disease Control and Prevention, 2009) via one-step RT-PCR. Amplicons were Sanger-sequenced by Sangon Biotech (Shanghai, China). Full-genome were generated using the SuperScript™ IV First Strand Synthesis System and Second-strand cDNA synthesis kit (Invitrogen, USA) following the manufacturer's protocol. Sequencing was performed using next-generation sequencing platforms such as the Illumina sequencing platform. Raw reads were assembled using the CLC Genomics Workbench. A total of 663 sequences were identified during the study period and used for the temporal distribution analysis of the three clusters. Viral genotype was determined by phylogenetic clustering based on the VP1 region (Zhang et al., 2010).

### 2.4 Phylogenetic analysis

Reference sequences from NCBI (Supplementary Table 1), along with 352 CVA16 VP1 sequences and 62 full-length sequences from Fujian Province, which were selected as representative sequences based on sampling date and location, were used to construct VP1 and full-length genome phylogenetic trees. For different genomic regions, we constructed phylogenetic trees for the 5′ untranslated region (5′ UTR), the structural protein region (P1), the non-structural protein regions (P2 and P3), and 3′ untranslated region (3′ UTR). To determine the optimal nucleotide substitution model for subsequent phylogenetic analyses, jModelTest 2.1.7 (Darriba et al., 2012) was used. Phylogenetic analyses were performed in MEGA 12.0 (Kumar et al., 2024) using the GTR+G+I model with 1,000 bootstrap replicates, and visualized by iTOL (https://itol.embl.de). The geographic and temporal distributions of the deposited sequences are summarized in Supplementary Table 2 and Supplementary Figure 1.

### 2.5 Evolutionary analysis

To estimate tMRCA, substitution rates, and population dynamics, the VP1 sequences dataset (only sequences from Fujian Province) was screened using TempEst v1.5.3 (Rambaut et al., 2016) with the “root-to-tip” option to assess the temporal signal, after which it was analyzed using BEAST v1.10.4. The analysis employed identical settings: the GTR+G+I model, a strict molecular clock, and a Bayesian Skygrid model. The Markov Chain Monte Carlo (MCMC) chain was run for 200 million generations with sampling every 20, 000 generations. Additionally, each of the three clusters (B1a-c) within the total dataset was analyzed separately. Parameters were evaluated for convergence, and ESS values (>200) were confirmed using Tracer v1.7.2 (Drummond et al., 2012; Rambaut et al., 2018). Finally, a Maximum Clade Credibility (MCC) tree was summarized via TreeAnnotator v1.10.4 (10% burn-in) and visualized using Fig Tree v1.4.4 (http://tree.bio.ed.ac.uk/software/figtree/). Bayesian Skygrid plots were constructed by Tracer v1.7.2 showing the effective population size over time.

## 3 Results

### 3.1 Epidemiological characteristics of HFMD in Fujian Province

**Figure 1:**
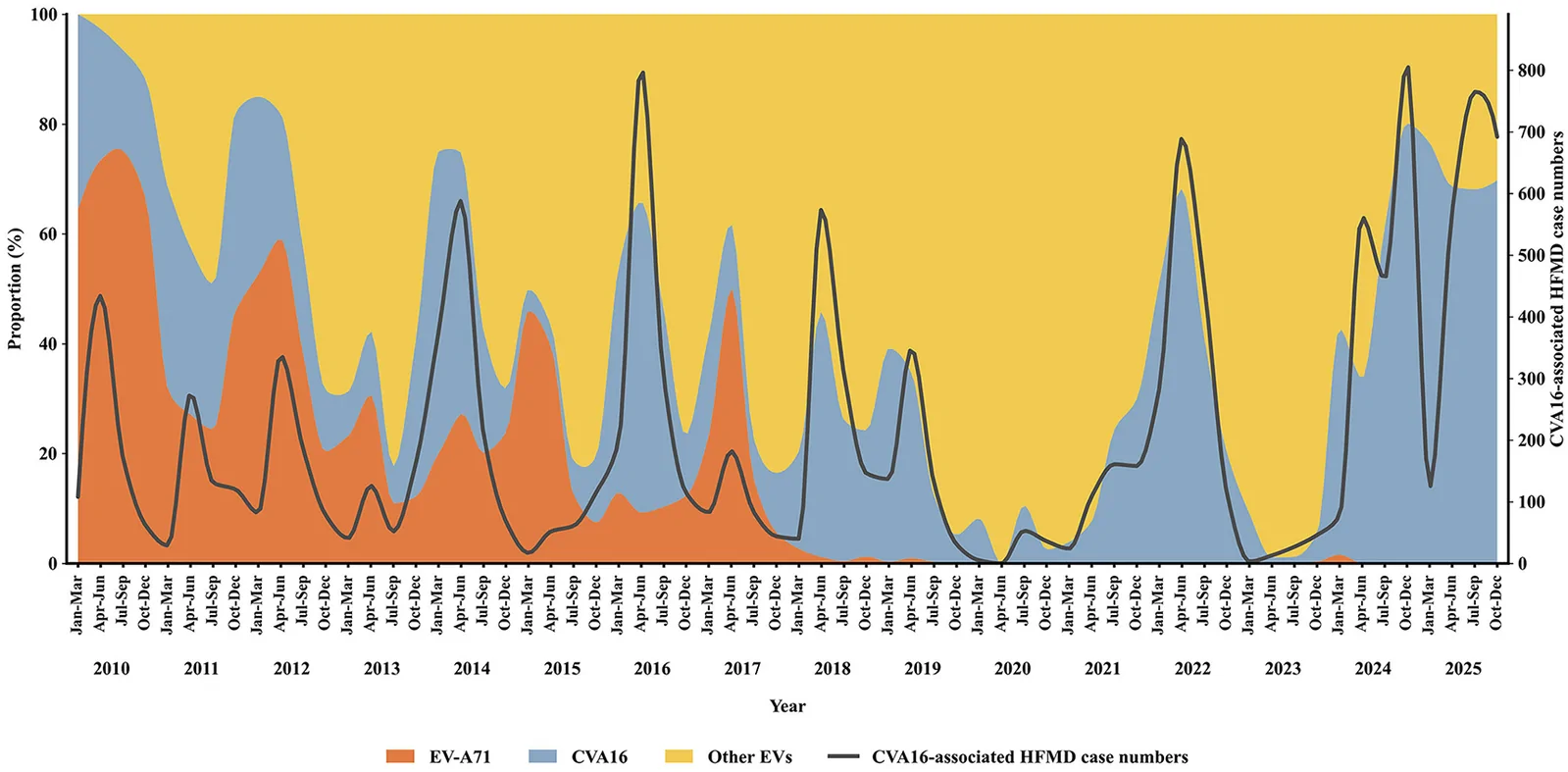
Pathogen Spectrum Composition of HFMD in Fujian Province from 2010 to 2025. Percentage stacked bars represent the quarterly percentage of EV-A71, CVA16, and other EVs among confirmed HFMD cases. The overlaid curve shows the number of CVA16-associated HFMD cases during the period.

From 2010 to 2025, a total of 52,530 laboratory-confirmed HFMD cases were included. The primary pathogens identified were EV-A71, CVA16, and other enteroviruses (defined as non-EV-A71 and non-CVA16; hereinafter “other EVs”). The annual shift in the pathogen spectrum is illustrated in Figure 1. The proportion of EV-A71 decreased progressively from its peak in 2010, falling to negligible levels after 2019. In contrast, the proportion of other EVs rose consistently from 2010 onward and became the dominant pathogen. During 2010–2025, CVA16 accounted for 25.96% (13639/52530) of cases. Both the proportion and case numbers of CVA16 exhibited biennial fluctuations, increasing in even-numbered years and decreasing in odd-numbered years. Since 2024, CVA16 has has overtaken other EVs to emerge as the most predominant pathogen.

The epidemic trend of CVA16-associated HFMD exhibited a biennial periodicity, with higher incidence in even-numbered years. Cases numbers began to rise in late autumn/winter (October–December) of odd-numbered years, peaked in spring–summer (January–June) of the subsequent even-numbered year, and then declined. This pattern indicates that CVA16 circulation showed a spring–summer predominance, however, it changed after 2023, when the seasonal peak transitioned toward the autumn–winter.

### 3.2 Spatial distribution of CVA16-associated HFMD in Fujian Province

**Figure 2:**
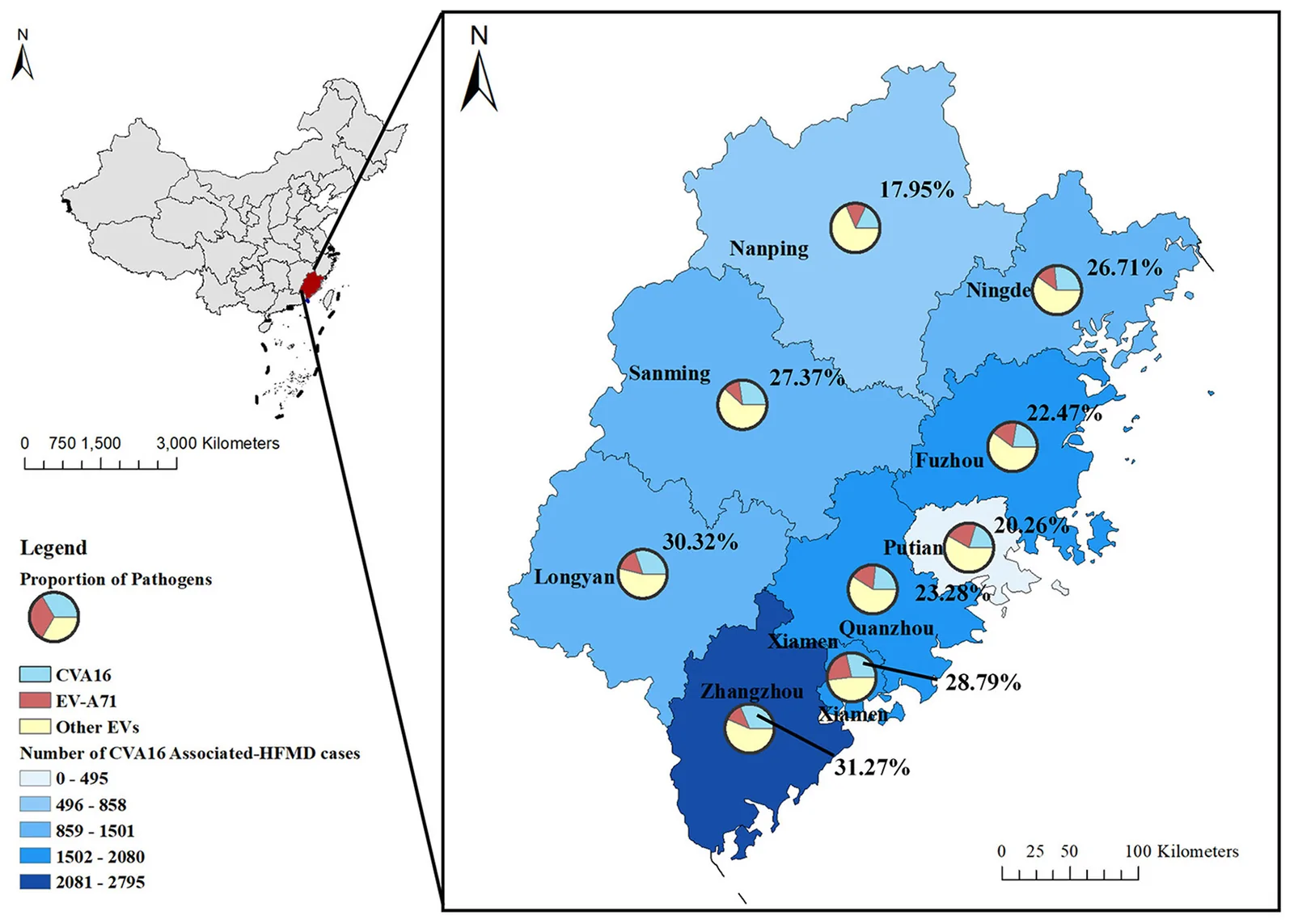
HFMD epidemiological regions and distribution in Fujian Province from 2010 to 2025. The base map is based on the standard map (No. GS(2024)0650) provided by the National Platform for Common Geospatial Information Services (https://www.tianditu.gov.cn/). The original boundary of the map has not been modified. The pie charts show the pathogen composition in each city. A color gradient from light blue to dark blue indicates the number of CVA16-associated HFMD cases.

Fujian Province is located on the southeastern coast of mainland China. It has multiple ports, frequent trade activities, and substantial population movement. It is also a major hometown of overseas Chinese. Over the 16-year study period, other EVs constituted the predominant pathogen across all cities in the province. CVA16-associated HFMD cases were mainly concentrated in southern cities. Among these areas, Zhangzhou city reported both the highest cumulative case count and the largest relative proportion of infections, suggesting it as a major cluster area for CVA16 outbreaks within the province (Figure 2).

### 3.3 Demographic distribution of CVA16-associated HFMD in Fujian Province

**Figure 3:**
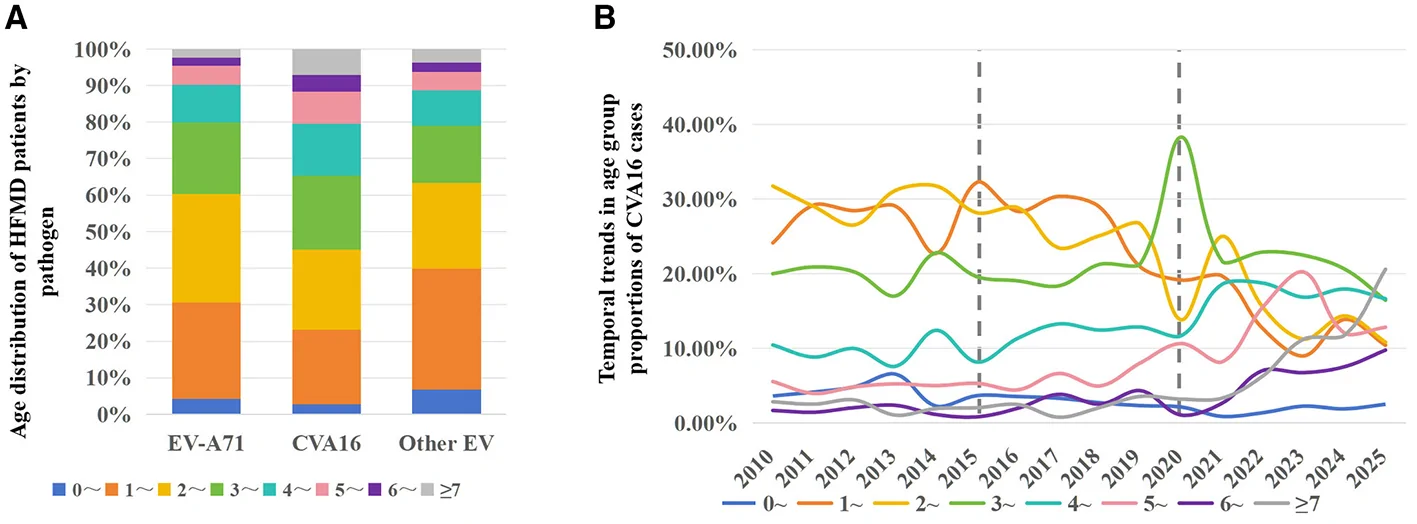
The Age Distribution of HFMD in Fujian Province from 2010 to 2025. **(A)** Age distribution of HFMD patients by pathogen. Stacked bar chart showing the percentage distribution of age groups among patients infected with different enteroviruses (EV-A71, CVA16, and other EVs) in Fujian Province. **(B)** Temporal trends in the proportion of age group among CVA16-associated HFMD cases.

From 2010 to 2025, the male-to-female ratio of CVA16-associated HFMD cases in Fujian Province was 1.69:1. The age distribution of HFMD patients is shown in Figure 3A. Children aged 1–3 years were the predominant group; however, the proportion of CVA16 patients aged ≥4 years was higher than that of EV-A71 and other EVs. As shown in Figure 3B, the proportions of the 1- and 2-year age groups were the highest during 2010–2019 but gradually declined after 2019. The proportion of the 3-year age group remained high after 2020, while the proportion of children aged ≥4 years progressively increased. Finer strata of the ≥7 years age group is provided in Supplemental Figure 2.

### 3.4 Phylogenetic and phylodynamic analysis of CVA16 in Fujian Province

#### 3.4.1 Lineage dynamics of CVA16 B1 subgenotype in Fujian Province

**Figure 4:**
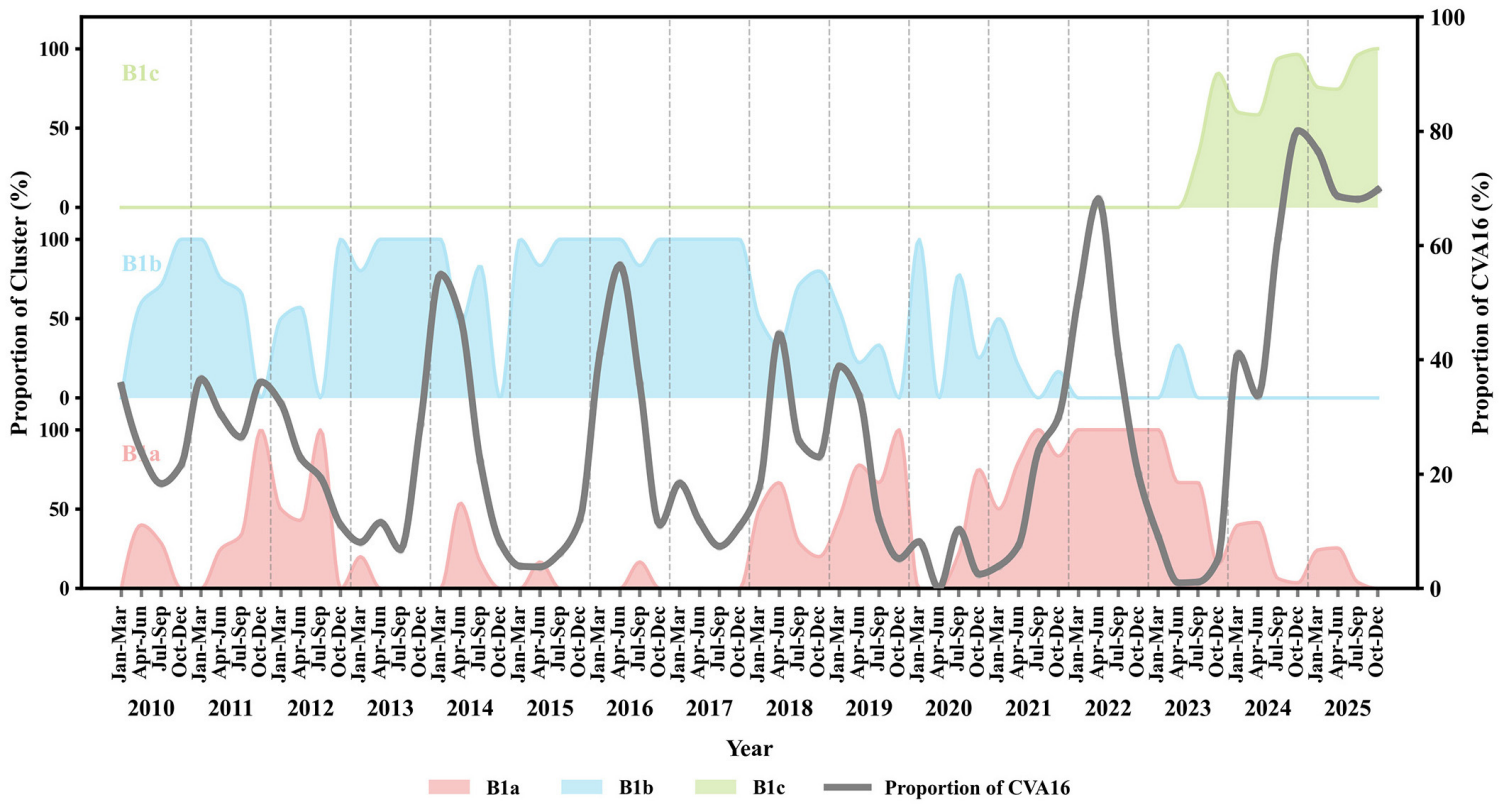
The Temporal distribution of three clusters(B1a–c) of CVA16 in Fujian Province from 2010 to 2025. The overlaid curve indicates the proportion of laboratory-confirmed CVA16 cases during the period.

From 2010 to 2025, the CVA16 strains associated with HFMD in Fujian Province were indentified as subgenotype B1, which comprised three evolutionary clusters: B1a, B1b, and B1c (Figure 4). The lineage transition progressed through two phases. During the first phase (2010–2022), cluster B1a and B1b co-circulated: before 2018, B1b was the dominant, which shifted to B1a after 2018. In the second phase (2023–2025), B1a and B1c co-circulated, with B1c, which emerged in the second half of 2023, becoming the dominant.

#### 3.4.2 Phylogenetic and phylodynamic analysis based on VP1 Region

**Figure 5:**
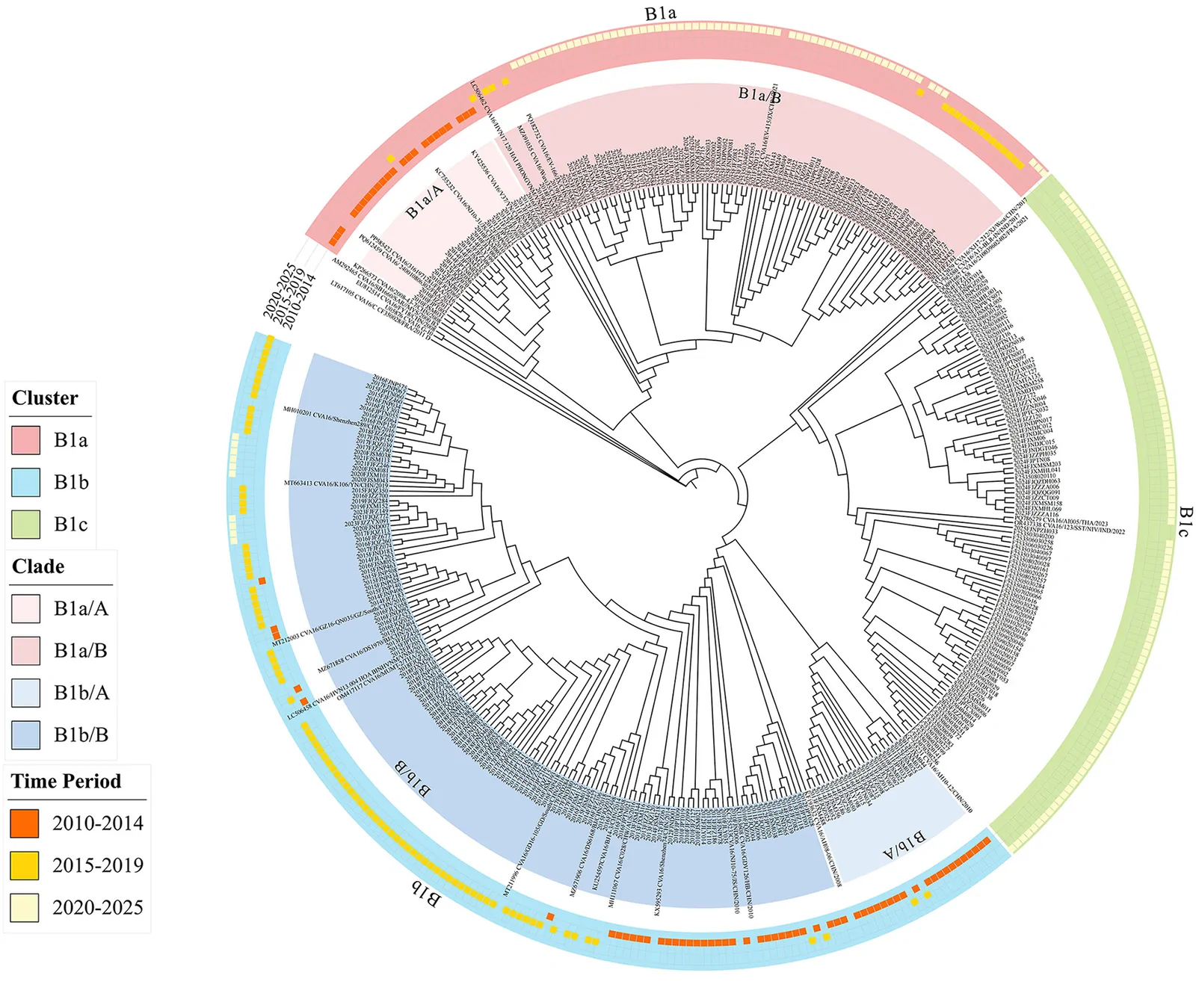
The Maximum likelihood phylogenetic tree based on the VP1 region (891 bp). A total of 385 sequences were analyzed, comprising 352 sequences obtained in this study and 33 reference sequences retrieved from NCBI. The phylogenetic tree was constructed using MEGA12 under the maximum likelihood (ML) method, with bootstrap analysis performed using 1,000 replicates. Reference sequences representing all known CVA16 genotypes and subgenotypes were included. Fujian strains and reference sequences were color-coded as indicated in the legend.

CVA16 circulating in Fujian Province clustered together with contemporaneous strains from other regions of mainland China basing on the phylogenetic tree (Figure 5). Within the B1a and B1b, two different clusters were observed, hereinafter referred to as clade A and clade B, respectively, as operational labels only. These clades exhibited divergent evolutionary patterns: for B1a, a transition from clade A to clade B occurred around 2016, after which only B1a/B was detected. In contrast, B1b/A and B1b/B initially co-circulated; however, B1b/A was last detected earlier, while B1b/B persisted until 2023 before detection ceased. The cluster B1c was first detected in our local surveillance in Fujian Province in 2023, subsequently spread and caused cluster outbreaks. This cluster was phylogenetically closely related to a B1c strain reported in India in 2022 (GenBank accession: OR437338).

In this study, the evolutionary rate and the time to the most recent common ancestor (tMRCA) of the CVA16 subgenotype B1 VP1 region in Fujian Province were estimated using BEAST (Table 1). The substitution rate for subgenotype B1 was 4.35 × 10^−3^ substitutions per site per year (s/s/y), with a 95% highest posterior density (HPD) interval of 3.82–4.88 × 10^−3^ s/s/y. The evolutionary rate of the B1b exhibited a relatively faster rate compared to the other two clades.

**Table 1:** Time to the most recent common ancestor (tMRCA) and substitution rates of CVA16 lineages in Fujian Province.

Subgenotype	tMRCA	95% HPD interval	Substitution rate (s/s/y)	95% HPD interval (s/s/y)
B1	1995.71	1992.75, 1998.56	4.35 × 10^−3^	3.82 × 10^−3^, 4.88 × 10^−3^
B1a	2002.91	2000.82, 2004.98	4.30 × 10^−3^	3.53 × 10^−3^, 5.06 × 10^−3^
B1b	2003.54	2001.76, 2005.35	4.55 × 10^−3^	3.90 × 10^−3^, 5.23 × 10^−3^
B1c	2012.90	2007.64, 2017.54	3.08 × 10^−3^	1.90 × 10^−3^, 4.21 × 10^−3^

**Figure 6:**
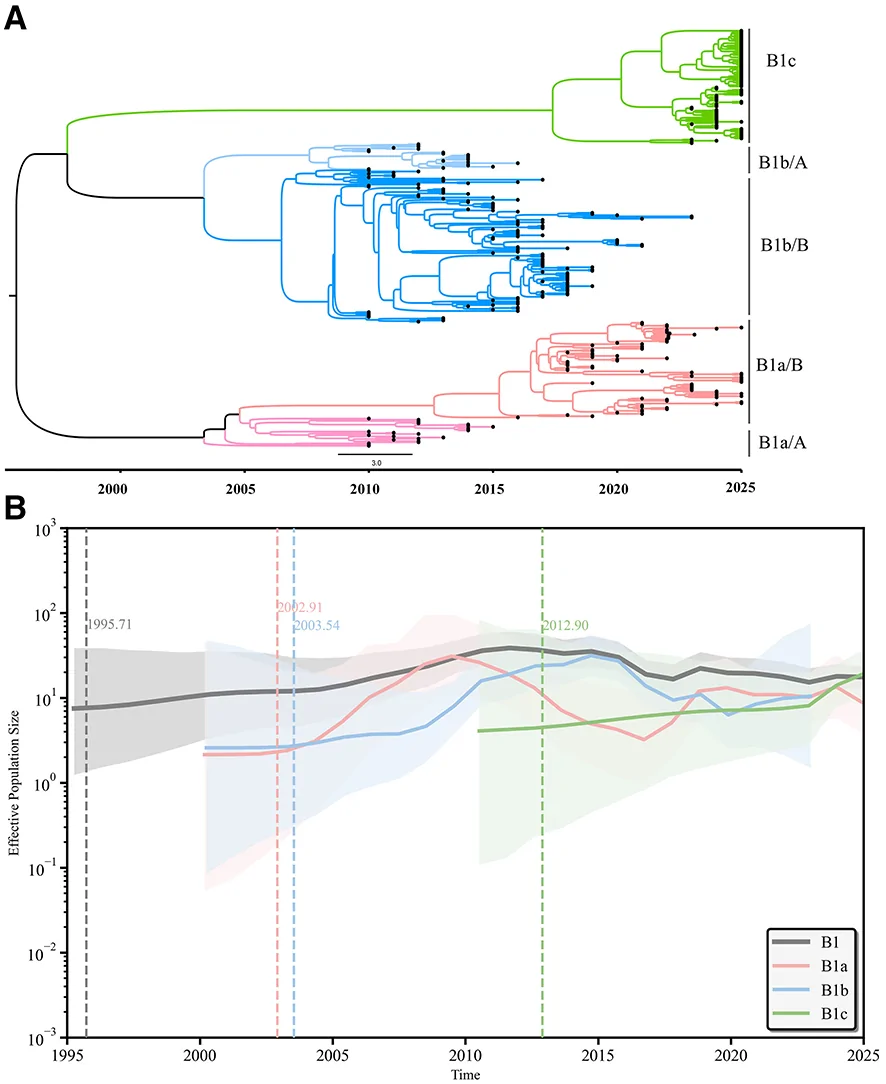
Epidemiological and population dynamics of CVA16 in Fujian Province. **(A)** Time-scaled maximum clade credibility(MCC) tree based on VP1 region (891 bp) using BEAST v1.10.4. **(B)** Bayesian Skygrid plot showing the effective population size over time. The solid lines represent the median estimates, and the shaded areas indicate the 95% highest posterior density (HPD) intervals.

The MCC tree topology was largely congruent with the ML tree (Figure 6A), revealing a ladder-like structure indicative of the continuous circulation and broad geographic expansion of CVA16 subgenotype B1 across Fujian Province from 2010 to 2025. The Skygrid plot showed that the effective population size of CVA16 B1 in Fujian Province remained generally stable, with a slight increasing trend, then a modest decline around 2015 to 2020 and minor fluctuations to date (Figure 6B).

The three clusters exhibited reciprocal population dynamics, characterized by asynchronous expansions and contractions, suggesting potential ecological competition among them. The Skygrid analysis indicates that the effective population size of B1c exhibits a continuous upward trend and has yet to reach a plateau, suggesting its expansion may be ongoing.

#### 3.4.3 Whole-genome analysis of CVA16

**Figure 7:**
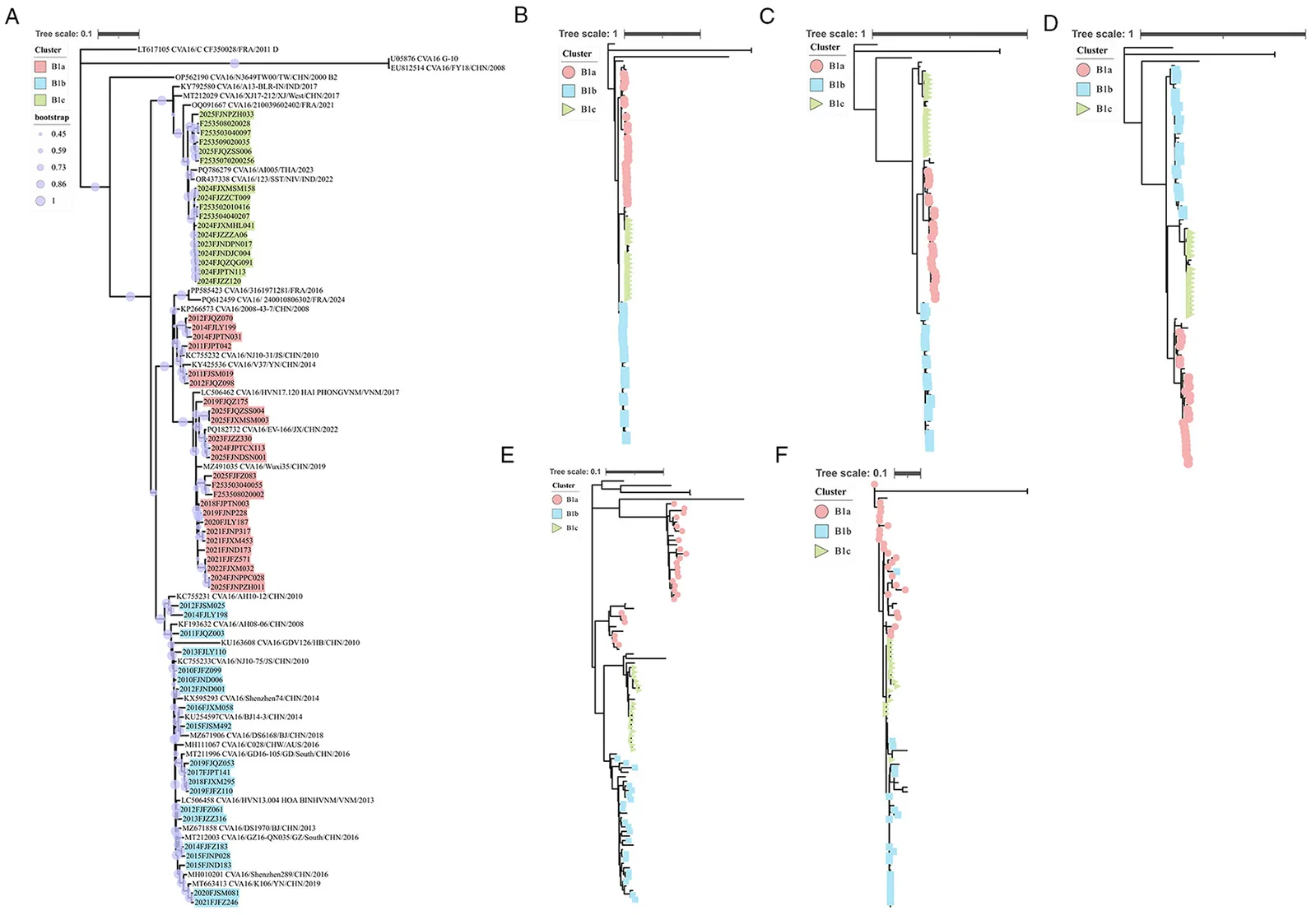
Phylogenetic trees based on the complete genome and different genomic regions of CVA16 strains from Fujian Province. Each tree was constructed using 93 sequences, including 62 sequences obtained in this study and 31 reference sequences retrieved from NCBI. The full-length sequences in the dataset ranged from 7,313 to 7,457 bp. Phylogenetic reconstruction was performed using MEGA12 under the maximum likelihood (ML) method, with bootstrap support assessed via 1,000 replicates. Genomic region boundaries were defined based on the CVA16 strain G-10 (GenBank accession: U05876). **(A)** In the complete genome tree, the Fujian strains were color-coded according to their respective clades. In the trees for specific genomic regions **(B–F)**, the Fujian strains were distinguished by different symbols as indicated in the figure legend. **(B)** P1 region; **(C)** P2 region; **(D)** P3 region; **(E)** 5' untranslated region (5'UTR); **(F)** 3' untranslated region (3'UTR).

A total of 62 complete CVA16 genome sequences were subjected to analysis, comprising B1a (*n* = 25), B1b (*n* = 20), B1c (*n* = 17). Phylogenetic analysis was performed alongside 31 reference sequences retrieved from NCBI. All obtained sequences clustered into subgenotype B1 (Figure 7A). The phylogenetic trees constructed from the P1, P2 and P3 regions (Figures 7B–D) were topologically congruent with the tree generated from the full-length genome sequences, indicating that the genetic characteristics of the protein-coding regions are relatively stable within the B1 subgenotype. The phylogenetic tree based on the 5′UTR and 3′UTR (Figures 7E, F) showed that the three clades of the B1 subgenotype in Fujian Province formed distinct, independent branches with significant internal clustering. While B1a formed a standalone cluster, one B1b strain grouped within the B1a cluster based on the 3′UTR. Although this discrepancy initially suggested a potential recombination event, however, no recombination signal was confirmed by SimPlot analysis, which may be attributed to the short length of the 3′UTR region.

## 4 Discussion

The Asia-Pacific region is a high incidence area of EV-related diseases, with frequent outbreaks of HFMD (Gopalkrishna and Ganorkar, 2021; Nhu et al., 2021; Liu et al., 2025). During the Coronavirus Disease 2019 (COVID-19) pandemic, the implementation of stringent non-pharmaceutical interventions (NPIs) in mainland China led to a significant decline in the annual reported incidence of HFMD (Zhou et al., 2023). The incidence subsequently rebounded to 118.7077 cases per 100,000 (National Disease Control and Prevention Administration, 2024), ranking among the top five of the national notifiable infectious diseases of Class C infectious diseases in 2023. The southeastern coastal region serves as the epicenter of HFMD aggregation and transmission in mainland China (Han et al., 2020), with transmission capacity exhibiting a decreasing gradient from southeast to northwest (Zheng et al., 2025). As an integral part of this transmission epicenter, Fujian plays a decisive role in seeding and sustaining the diffusion of HFMD in China. Based on the surveillance data from the Fujian provincial laboratory-based surveillance network for HFMD, this study leveraged a 16-year longitudinal analysis to investigate the epidemiological characteristics, population dynamics, lineage reshuffling, and the impact of the introduction and circulation of novel CVA16 cluster on the prevalence of HFMD in Fujian Province.

This study reveals changes in the epidemiological patterns of HFMD in Fujian Province. First, the predominant pathogen has shifted. Between 2011 and 2023, other EV were the leading pathogens, followed by CVA16. After 2023, the predominant pathogen transitioned to CVA16, a trend consistent with that reported in mainland China (Sun et al., 2023; Zeng et al., 2025). Second, the age distribution of CVA16-associated HFMD cases changed after 2020, with an increasing proportion of older children over time. These phenomena may be attributed to factors such as changes in the pathogen spectrum, climate conditions, and the accumulation of susceptible populations resulting from NPIs implemented during the COVID-19 pandemic (Peng et al., 2022; Zhao et al., 2022; Lau et al., 2024; Bednarska et al., 2025). Third, the proportion of CVA16 remained high during 2024–2025, departing from the previous biennial pattern. This persistent high level may be explained by Yan et al. (2026): following NPIs, the enterovirus serotypes with higher transmissibility (*R*_0_) and therefore a younger mean age at infection experience a bigger reduction in transmission. In Fujian, however, the age distribution of CVA16 infection has an increasing proportion of CVA16 cases was observed in the ≥7 years age group—a pattern consistent with a relatively smaller reduction in transmission, which would allow CVA16 to remain at a high proportion.

The pathogen spectrum composition plays a role in shaping the seasonal epidemic patterns of HFMD (Hou et al., 2026). According to the findings of this study, the fluctuations in the proportion of CVA16 and the shift in its epidemic season may be related to the replacement dynamics among different evolutionary lineages. Historically, the CVA16 prevalent in mainland China from 2000 to 2018 were predominantly of the B1 subgenotype, with B1b being the leading cluster amid the co-circulation with B1a (Han et al., 2020). However, a shift occurred in Guangdong Province, where B1a replaced B1b as the dominant subgenotype after 2019, followed by the emergence of B1c in 2023, which established absolute dominance by 2024 (Zeng et al., 2025). Our study indicates that the shift in dominant CVA16 lineage in Fujian Province aligned with the national trends. While B1a replaced B1b as the primary cluster after 2019, its dominance was swiftly challenged by the emergence of B1c, which rapidly took over and has maintained its dominance for two consecutive years. The B1c was prevalent primarily in India (Ganorkar et al., 2017; Tikute and Lavania, 2023) and only sporadic cases had been reported in mainland China, with no evidence of local transmission before 2022 (Han et al., 2020; Hu et al., 2021). Since 2023, however, some countries have reported both imported and local cases of B1c (Dumaidi et al., 2025; Taoma et al., 2025). The B1c currently circulating in Fujian Province and other areas of China shares high similarity with the Indian strains (Yang et al., 2025). This, along with possible gaps in population immunity, suggests that B1c likely possesses enhanced transmissibility, allowing it to become the dominant sublineage in a short time.

The effective population size of CVA16 exhibited a generally stable yet fluctuating trend over the past 16 years. However, the diverging population dynamics among the three clusters suggest potential competitive displacement between them. We propose that the period from 2015 to 2020 may have served as a critical epidemiological transition point for CVA16 in Fujian Province. During this period, clade B1a/A and B1b/A remained undetected. Meanwhile, the two clades within B1a exhibited a certain degree of genetic distance from each other, raising the possibility that they may have been reintroduced from external sources rather than descending from a shared local ancestor. This “appearance–disappearance–reappearance” cyclical pattern in B1a also observed in Shenyang, Liaoning Province (Li et al., 2024). It may be driven by viral mutation, natural selection pressures, or immune evasion (Hu et al., 2021; Han et al., 2023; Anjum et al., 2024; Mo et al., 2026). Additionally, pathogens may also influence each other during co-circulation (Zhou et al., 2023). Furthermore, no significant signs of recombination were detected in our sequences. Thus, in addition to genetic recombination, another scenario worth considering is that previously dormant or sporadic lineages may become reactivated under favorable conditions, thereby dominating the outbreak (Li et al., 2024). Admittedly, the possibility of other mechanisms cannot be ruled out. These findings suggest that the public health threat posed by enteroviruses is substantially underestimated. Although our findings characterize the epidemiology of CVA16, several limitations should be noted. Our data comes from Fujian province, so the results may not apply to other regions. Furthermore, variations in economic development and testing preferences across regions might lead to bias, although this does not affect the robustness of our main conclusions. The lack of laboratory or animal experiments limits our understanding of the pathogenesis of CVA16, highlighting the need for further studies. Additionally, the clinical associations between viral genotypes and disease manifestations warrant further investigation.

## 5 Conclusion

In summary, the B1 subgenotype of CVA16 was one of the primary pathogens of HFMD in Fujian Province. Its three evolutionary lineages, cluster B1a, B1b, and B1c, have shown alternating patterns of dominance, among which the newly emerged B1c cluster surpassed the others to become the predominant strain after 2023, triggering the epidemic.

## Data Availability

The datasets presented in this study can be found in online repositories. The names of the repository/repositories and accession number(s) can be found below: https://www.ncbi.nlm.nih.gov/genbank/, KU595959–KU595963, KU595966–KU595969, KU595975–KU595991, KU595993–KU596003, KU596007, KU596009, KU596012–KU596016, KU596018–KU596019, and KU596021–KU596022. https://nmdc.cn/, NMDCN000CVLF–NMDCN000CVT7, NMDCN000CVLC–NMDCN000CVLE, NMDCN000CVJG–NMDCN000CVLB.
