# Supplementary material for: Spatiotemporal dynamics and genotypic shift of Coxsackievirus A16 in Fujian, China, from 2010 to 2025
Source: Front Microbiol. 2026 Aug 11;17:1893929. doi: 10.3389/fmicb.2026.1893929 (PMC13503587; doi:10.3389/fmicb.2026.1893929)
Supplement: Supplementary file 1 [file Table_1.doc]

Supplementary Material

**Table S1. GenBank accession numbers for the reference sequences involved in this study.**

| GenBank number | Isolate | Country | Year | Genotype |
| --- | --- | --- | --- | --- |
| PQ612459 | 240010806302 | FRA | 2024 | B1a |
| PQ182742 | EV-415/JX | CHN | 2021 | B1a |
| PQ182732 | EV-166/JX | CHN | 2022 | B1a |
| PP585423 | 3161971281 | FRA | 2016 | B1a |
| OM417117 | MUMT-2 | THA | 2016 | B1b |
| MZ671906 | DS6168/BJ | CHN | 2018 | B1b |
| MZ671858 | DS1970/BJ | CHN | 2013 | B1b |
| MZ491035 | Wuxi35 | CHN | 2019 | B1a |
| MT663413 | K106/YN | CHN | 2019 | B1b |
| MT212003 | GZ16-QN035/GZ/South | CHN | 2016 | B1b |
| MT211996 | GD16-105/GD/South | CHN | 2016 | B1b |
| KY792580 | A13-BLR-IN | IND | 2017 | B1c |
| MT212029 | XJ17-212/XJ/West | CHN | 2017 | B1c |
| OQ091667 | 210039602402 | FRA | 2021 | B1c |
| OR437338 | 123/SST/NIV | IND | 2022 | B1c |
| PQ786279 | AI005 | THA | 2023 | B1c |
| MH111067 | C028/CHW | AUS | 2016 | B1b |
| MH010201 | Shenzhen289 | CHN | 2016 | B1b |
| LC506462 | HVN17.120_HAI_PHONGVNM | VNM | 2017 | B1a |
| LC506458 | HVN13.004_HOA_BINHVNM | VNM | 2013 | B1b |
| KY425536 | V37/YN | CHN | 2014 | B1a |
| KX595293 | Shenzhen74 | CHN | 2014 | B1b |
| KU254597 | BJ14-3 | CHN | 2014 | B1b |
| KU163608 | GDV126/HB | CHN | 2010 | B1b |
| KP266573 | 2008-43-7 | CHN | 2008 | B1a |
| KF193632 | AH08-06 | CHN | 2008 | B1b |
| KC755233 | NJ10-75/JS | CHN | 2010 | B1b |
| KC755232 | NJ10-31/JS | CHN | 2010 | B1a |
| KC755231 | AH10-12 | CHN | 2010 | B1b |
| EU812514 | FY18 | CHN | 2008 | A |
| U05876 | G-10 | ZAF | 1951 | A |
| LT617105 | CF350028 | FRA | 2011 | D |
| AM292465 | SB1660/SAR/MYS/2000 B | MYS | 2000 | B2 |
| OP562190 | N3649TW00 | CHN | 2000 | B2 |

**Table S2. Information about the Sequences of CVA16 from Fujian in this study.**

| Isolate | GenBank accession | NMDC accession numbers | Usage | City | Year |
| --- | --- | --- | --- | --- | --- |
| 2010FJFZ099 |  | NMDCN000CVLB | Whole genome/VP1 phylogenetic tree | Fuzhou | 2010 |
| 2010FJFZ127 | KU595959 |  | VP1 phylogenetic tree | Fuzhou | 2010 |
| 2010FJLY007 |  | NMDCN000CVNU | VP1 phylogenetic tree | Longyan | 2010 |
| 2010FJLY035 |  | NMDCN000CVNV | VP1 phylogenetic tree | Longyan | 2010 |
| 2010FJND006 |  | NMDCN000CVLA | Whole genome/VP1 phylogenetic tree | Ningde | 2010 |
| 2010FJPT001 |  | NMDCN000CVO0 | VP1 phylogenetic tree | Putian | 2010 |
| 2010FJPT009 |  | NMDCN000CVO1 | VP1 phylogenetic tree | Putian | 2010 |
| 2010FJPT019 | KU595961 |  | VP1 phylogenetic tree | Putian | 2010 |
| 2010FJQZ002 | KU595963 |  | VP1 phylogenetic tree | Quanzhou | 2010 |
| 2010FJQZ067 |  | NMDCN000CVO2 | VP1 phylogenetic tree | Quanzhou | 2010 |
| 2010FJSM021 |  | NMDCN000CVO3 | VP1 phylogenetic tree | Sanming | 2010 |
| 2010FJSM027 | KU595962 |  | VP1 phylogenetic tree | Sanming | 2010 |
| 2010FJSM038 |  | NMDCN000CVO4 | VP1 phylogenetic tree | Sanming | 2010 |
| 2010FJLY004 |  | NMDCN000CVR3 | VP1 phylogenetic tree | Longyan | 2010 |
| 2010FJPT002 |  | NMDCN000CVR4 | VP1 phylogenetic tree | Putian | 2010 |
| 2010FJQZ069 |  | NMDCN000CVR5 | VP1 phylogenetic tree | Quanzhou | 2010 |
| 2010FJSM031 |  | NMDCN000CVR6 | VP1 phylogenetic tree | Sanming | 2010 |
| 2010FJXM005 | KU595960 |  | VP1 phylogenetic tree | Xiamen | 2010 |
| 2010FJZZ011 |  | NMDCN000CVR7 | VP1 phylogenetic tree | Zhangzhou | 2010 |
| 2011FJPT033 |  | NMDCN000CVO5 | VP1 phylogenetic tree | Putian | 2011 |
| 2011FJQZ004 | KU595966 |  | VP1 phylogenetic tree | Quanzhou | 2011 |
| 2011FJQZ006 | KU595967 |  | VP1 phylogenetic tree | Quanzhou | 2011 |
| 2011FJQZ007 | KU595968 |  | VP1 phylogenetic tree | Quanzhou | 2011 |
| 2011FJSM018 |  | NMDCN000CVO6 | VP1 phylogenetic tree | Sanming | 2011 |
| 2011FJPT042 |  | NMDCN000CVL9 | Whole genome/VP1 phylogenetic tree | Putian | 2011 |
| 2011FJQZ011 | KU595969 |  | VP1 phylogenetic tree | Quanzhou | 2011 |
| 2011FJSM019 |  | NMDCN000CVL7 | Whole genome/VP1 phylogenetic tree | Sanming | 2011 |
| 2011FJQZ003 |  | NMDCN000CVL8 | Whole genome phylogenetic tree | Quanzhou | 2011 |
| 2012FJFZ051 | KU595972 |  | VP1 phylogenetic tree | Fuzhou | 2012 |
| 2012FJFZ061 |  | NMDCN000CVL6 | Whole genome/VP1 phylogenetic tree | Fuzhou | 2012 |
| 2012FJLY014 | KU595982 |  | VP1 phylogenetic tree | Longyan | 2012 |
| 2012FJND001 |  | NMDCN000CVL5 | Whole genome/VP1 phylogenetic tree | Ningde | 2012 |
| 2012FJPT020 |  | NMDCN000CVO7 | VP1 phylogenetic tree | Putian | 2012 |
| 2012FJPT028 | KU595976 |  | VP1 phylogenetic tree | Putian | 2012 |
| 2012FJSM025 |  | NMDCN000CVL2 | Whole genome/VP1 phylogenetic tree | Sanming | 2012 |
| 2012FJXM037 |  | NMDCN000CVO8 | VP1 phylogenetic tree | Xiamen | 2012 |
| 2012FJXM044 | KU595975 |  | VP1 phylogenetic tree | Xiamen | 2012 |
| 2012FJLY009 | KU595981 |  | VP1 phylogenetic tree | Longyan | 2012 |
| 2012FJFZ007 |  | NMDCN000CVR8 | VP1 phylogenetic tree | Fuzhou | 2012 |
| 2012FJFZ102 | KU595973 |  | VP1 phylogenetic tree | Fuzhou | 2012 |
| 2012FJND012 | KU595984 |  | VP1 phylogenetic tree | Ningde | 2012 |
| 2012FJNP005 | KU595980 |  | VP1 phylogenetic tree | Nanping | 2012 |
| 2012FJQZ056 | KU595979 |  | VP1 phylogenetic tree | Quanzhou | 2012 |
| 2012FJQZ070 |  | NMDCN000CVL4 | Whole genome/VP1 phylogenetic tree | Quanzhou | 2012 |
| 2012FJQZ098 |  | NMDCN000CVL3 | Whole genome/VP1 phylogenetic tree | Quanzhou | 2012 |
| 2013FJFZ098 | KU595985 |  | VP1 phylogenetic tree | Fuzhou | 2013 |
| 2013FJFZ118 | KU595986 |  | VP1 phylogenetic tree | Fuzhou | 2013 |
| 2013FJFZ186 |  | NMDCN000CVO9 | VP1 phylogenetic tree | Fuzhou | 2013 |
| 2013FJLY110 |  | NMDCN000CVL1 | Whole genome/VP1 phylogenetic tree | Longyan | 2013 |
| 2013FJNP078 |  | NMDCN000CVOA | VP1 phylogenetic tree | Nanping | 2013 |
| 2013FJQZ120 |  | NMDCN000CVOB | VP1 phylogenetic tree | Quanzhou | 2013 |
| 2013FJQZ215 |  | NMDCN000CVOC | VP1 phylogenetic tree | Quanzhou | 2013 |
| 2013FJQZ381 | KU595988 |  | VP1 phylogenetic tree | Quanzhou | 2013 |
| 2013FJSM344 | KU595989 |  | VP1 phylogenetic tree | Sanming | 2013 |
| 2013FJXM316 |  | NMDCN000CVOD | VP1 phylogenetic tree | Xiamen | 2013 |
| 2013FJXM488 | KU595991 |  | VP1 phylogenetic tree | Xiamen | 2013 |
| 2013FJZZ013 | KU595990 |  | VP1 phylogenetic tree | Zhangzhou | 2013 |
| 2013FJZZ316 |  | NMDCN000CVL1 | Whole genome/VP1 phylogenetic tree | Zhangzhou | 2013 |
| 2013FJZZ459 |  | NMDCN000CVOE | VP1 phylogenetic tree | Zhangzhou | 2013 |
| 2013FJNP008 | KU595987 |  | VP1 phylogenetic tree | Nanping | 2013 |
| 2014FJFZ183 |  | NMDCN000CVKV | Whole genome/VP1 phylogenetic tree | Fuzhou | 2014 |
| 2014FJFZ227 |  | NMDCN000CVOF | VP1 phylogenetic tree | Fuzhou | 2014 |
| 2014FJLY186 | KU595993 |  | VP1 phylogenetic tree | Longyan | 2014 |
| 2014FJLY198 |  | NMDCN000CVLC | Whole genome/VP1 phylogenetic tree | Longyan | 2014 |
| 2014FJLY263 | KU595995 |  | VP1 phylogenetic tree | Longyan | 2014 |
| 2014FJNP406 | KU595996 |  | VP1 phylogenetic tree | Nanping | 2014 |
| 2014FJPTN034 | KU595999 |  | VP1 phylogenetic tree | Fuzhou | 2014 |
| 2014FJQZ025 | KU596007 |  | VP1 phylogenetic tree | Quanzhou | 2014 |
| 2014FJQZ250 | KU596002 |  | VP1 phylogenetic tree | Quanzhou | 2014 |
| 2014FJQZ339 | KU596003 |  | VP1 phylogenetic tree | Quanzhou | 2014 |
| 2014FJSM050 | KU596012 |  | VP1 phylogenetic tree | Sanming | 2014 |
| 2014FJSM052 | KU596013 |  | VP1 phylogenetic tree | Sanming | 2014 |
| 2014FJSM218 | KU596015 |  | VP1 phylogenetic tree | Sanming | 2014 |
| 2014FJSM356 | KU596009 |  | VP1 phylogenetic tree | Sanming | 2014 |
| 2014FJLY199 |  | NMDCN000CVKU | Whole genome/VP1 phylogenetic tree | Longyan | 2014 |
| 2014FJPTN028 | KU596001 |  | VP1 phylogenetic tree | Fuzhou | 2014 |
| 2014FJPTN032 | KU595998 |  | VP1 phylogenetic tree | Fuzhou | 2014 |
| 2014FJSM215 | KU596014 |  | VP1 phylogenetic tree | Sanming | 2014 |
| 2014FJSM226 | KU596016 |  | VP1 phylogenetic tree | Sanming | 2014 |
| 2014FJPTN031 |  | NMDCN000CVKT | Whole genome phylogenetic tree | Fuzhou | 2014 |
| 2015FJFZ460 |  | NMDCN000CVOG | VP1 phylogenetic tree | Fuzhou | 2015 |
| 2015FJND183 |  | NMDCN000CVKS | Whole genome/VP1 phylogenetic tree | Ningde | 2015 |
| 2015FJNP028 |  | NMDCN000CVKR | Whole genome/VP1 phylogenetic tree | Nanping | 2015 |
| 2015FJNP140 | KU596019 |  | VP1 phylogenetic tree | Nanping | 2015 |
| 2015FJNP434 |  | NMDCN000CVOH | VP1 phylogenetic tree | Nanping | 2015 |
| 2015FJNP463 |  | NMDCN000CVOI | VP1 phylogenetic tree | Nanping | 2015 |
| 2015FJNP508 |  | NMDCN000CVOJ | VP1 phylogenetic tree | Nanping | 2015 |
| 2015FJPT057 | KU596018 |  | VP1 phylogenetic tree | Putian | 2015 |
| 2015FJPTN060 |  | NMDCN000CVOK | VP1 phylogenetic tree | Fuzhou | 2015 |
| 2015FJQZ033 |  | NMDCN000CVOL | VP1 phylogenetic tree | Quanzhou | 2015 |
| 2015FJQZ087 |  | NMDCN000CVOM | VP1 phylogenetic tree | Quanzhou | 2015 |
| 2015FJQZ115 | KU596022 |  | VP1 phylogenetic tree | Quanzhou | 2015 |
| 2015FJQZ350 |  | NMDCN000CVON | VP1 phylogenetic tree | Quanzhou | 2015 |
| 2015FJQZ404 |  | NMDCN000CVOO | VP1 phylogenetic tree | Quanzhou | 2015 |
| 2015FJSM492 |  | NMDCN000CVKQ | Whole genome/VP1 phylogenetic tree | Sanming | 2015 |
| 2015FJFZ306 | KU596021 |  | VP1 phylogenetic tree | Fuzhou | 2015 |
| 2016FJFZ064 |  | NMDCN000CVOP | VP1 phylogenetic tree | Fuzhou | 2016 |
| 2016FJFZ082 |  | NMDCN000CVOQ | VP1 phylogenetic tree | Fuzhou | 2016 |
| 2016FJFZ106 |  | NMDCN000CVOR | VP1 phylogenetic tree | Fuzhou | 2016 |
| 2016FJFZ677 |  | NMDCN000CVOS | VP1 phylogenetic tree | Fuzhou | 2016 |
| 2016FJLY015 |  | NMDCN000CVOT | VP1 phylogenetic tree | Longyan | 2016 |
| 2016FJLY058 |  | NMDCN000CVOU | VP1 phylogenetic tree | Longyan | 2016 |
| 2016FJLY303 |  | NMDCN000CVOV | VP1 phylogenetic tree | Longyan | 2016 |
| 2016FJND092 |  | NMDCN000CVP0 | VP1 phylogenetic tree | Ningde | 2016 |
| 2016FJNP034 |  | NMDCN000CVP1 | VP1 phylogenetic tree | Nanping | 2016 |
| 2016FJNP120 |  | NMDCN000CVP2 | VP1 phylogenetic tree | Nanping | 2016 |
| 2016FJNP418 |  | NMDCN000CVP3 | VP1 phylogenetic tree | Nanping | 2016 |
| 2016FJNP575 |  | NMDCN000CVP4 | VP1 phylogenetic tree | Nanping | 2016 |
| 2016FJPT216 |  | NMDCN000CVP5 | VP1 phylogenetic tree | Putian | 2016 |
| 2016FJPTN021 |  | NMDCN000CVP6 | VP1 phylogenetic tree | Fuzhou | 2016 |
| 2016FJPTN055 |  | NMDCN000CVP7 | VP1 phylogenetic tree | Fuzhou | 2016 |
| 2016FJQZ020 |  | NMDCN000CVP8 | VP1 phylogenetic tree | Quanzhou | 2016 |
| 2016FJQZ260 |  | NMDCN000CVP9 | VP1 phylogenetic tree | Quanzhou | 2016 |
| 2016FJSM055 |  | NMDCN000CVPA | VP1 phylogenetic tree | Sanming | 2016 |
| 2016FJSM136 |  | NMDCN000CVPB | VP1 phylogenetic tree | Sanming | 2016 |
| 2016FJSM393 |  | NMDCN000CVPC | VP1 phylogenetic tree | Sanming | 2016 |
| 2016FJXM015 |  | NMDCN000CVPD | VP1 phylogenetic tree | Xiamen | 2016 |
| 2016FJXM058 |  | NMDCN000CVKP | Whole genome/VP1 phylogenetic tree | Xiamen | 2016 |
| 2016FJZZ523 |  | NMDCN000CVPE | VP1 phylogenetic tree | Zhangzhou | 2016 |
| 2016FJZZ598 |  | NMDCN000CVPF | VP1 phylogenetic tree | Zhangzhou | 2016 |
| 2016FJZZ700 |  | NMDCN000CVPG | VP1 phylogenetic tree | Zhangzhou | 2016 |
| 2016FJZZ595 |  | NMDCN000CVR9 | VP1 phylogenetic tree | Zhangzhou | 2016 |
| 2017FJFZ074 |  | NMDCN000CVPH | VP1 phylogenetic tree | Fuzhou | 2017 |
| 2017FJFZ098 |  | NMDCN000CVPI | VP1 phylogenetic tree | Fuzhou | 2017 |
| 2017FJFZ115 |  | NMDCN000CVPJ | VP1 phylogenetic tree | Fuzhou | 2017 |
| 2017FJFZ162 |  | NMDCN000CVPK | VP1 phylogenetic tree | Fuzhou | 2017 |
| 2017FJFZ322 |  | NMDCN000CVPL | VP1 phylogenetic tree | Fuzhou | 2017 |
| 2017FJFZ386 |  | NMDCN000CVPM | VP1 phylogenetic tree | Fuzhou | 2017 |
| 2017FJFZ423 |  | NMDCN000CVPN | VP1 phylogenetic tree | Fuzhou | 2017 |
| 2017FJLY882 |  | NMDCN000CVPO | VP1 phylogenetic tree | Longyan | 2017 |
| 2017FJND265 |  | NMDCN000CVPP | VP1 phylogenetic tree | Ningde | 2017 |
| 2017FJND284 |  | NMDCN000CVPQ | VP1 phylogenetic tree | Ningde | 2017 |
| 2017FJND380 |  | NMDCN000CVPR | VP1 phylogenetic tree | Ningde | 2017 |
| 2017FJNP067 |  | NMDCN000CVPS | VP1 phylogenetic tree | Nanping | 2017 |
| 2017FJNP159 |  | NMDCN000CVPT | VP1 phylogenetic tree | Nanping | 2017 |
| 2017FJPT034 |  | NMDCN000CVPU | VP1 phylogenetic tree | Putian | 2017 |
| 2017FJPT064 |  | NMDCN000CVPV | VP1 phylogenetic tree | Putian | 2017 |
| 2017FJPT141 |  | NMDCN000CVKO | Whole genome/VP1 phylogenetic tree | Putian | 2017 |
| 2017FJPT159 |  | NMDCN000CVQ0 | VP1 phylogenetic tree | Putian | 2017 |
| 2017FJQZ039 |  | NMDCN000CVQ1 | VP1 phylogenetic tree | Quanzhou | 2017 |
| 2017FJQZ062 |  | NMDCN000CVQ2 | VP1 phylogenetic tree | Quanzhou | 2017 |
| 2017FJQZ113 |  | NMDCN000CVQ3 | VP1 phylogenetic tree | Quanzhou | 2017 |
| 2017FJQZ379 |  | NMDCN000CVQ4 | VP1 phylogenetic tree | Quanzhou | 2017 |
| 2017FJZZ390 |  | NMDCN000CVQ5 | VP1 phylogenetic tree | Zhangzhou | 2017 |
| 2018FJFZ600 |  | NMDCN000CVQ6 | VP1 phylogenetic tree | Fuzhou | 2018 |
| 2018FJND089 |  | NMDCN000CVQ7 | VP1 phylogenetic tree | Ningde | 2018 |
| 2018FJND193 |  | NMDCN000CVQ8 | VP1 phylogenetic tree | Ningde | 2018 |
| 2018FJND463 |  | NMDCN000CVQ9 | VP1 phylogenetic tree | Ningde | 2018 |
| 2018FJNP590 |  | NMDCN000CVQA | VP1 phylogenetic tree | Nanping | 2018 |
| 2018FJQZ030 |  | NMDCN000CVQB | VP1 phylogenetic tree | Quanzhou | 2018 |
| 2018FJQZ080 |  | NMDCN000CVQC | VP1 phylogenetic tree | Quanzhou | 2018 |
| 2018FJQZ126 |  | NMDCN000CVQD | VP1 phylogenetic tree | Quanzhou | 2018 |
| 2018FJQZ393 |  | NMDCN000CVQE | VP1 phylogenetic tree | Quanzhou | 2018 |
| 2018FJQZ437 |  | NMDCN000CVQF | VP1 phylogenetic tree | Quanzhou | 2018 |
| 2018FJSM012 |  | NMDCN000CVQG | VP1 phylogenetic tree | Sanming | 2018 |
| 2018FJSM336 |  | NMDCN000CVQH | VP1 phylogenetic tree | Sanming | 2018 |
| 2018FJXM295 |  | NMDCN000CVKM | Whole genome/VP1 phylogenetic tree | Xiamen | 2018 |
| 2018FJXM514 |  | NMDCN000CVQI | VP1 phylogenetic tree | Xiamen | 2018 |
| 2018FJZZ327 |  | NMDCN000CVQJ | VP1 phylogenetic tree | Zhangzhou | 2018 |
| 2018FJZZ633 |  | NMDCN000CVQK | VP1 phylogenetic tree | Zhangzhou | 2018 |
| 2018FJZZ649 |  | NMDCN000CVQL | VP1 phylogenetic tree | Zhangzhou | 2018 |
| 2018FJZZ724 |  | NMDCN000CVQM | VP1 phylogenetic tree | Zhangzhou | 2018 |
| 2018FJFZ116 |  | NMDCN000CVRA | VP1 phylogenetic tree | Fuzhou | 2018 |
| 2018FJNP507 |  | NMDCN000CVRB | VP1 phylogenetic tree | Nanping | 2018 |
| 2018FJPTN003 |  | NMDCN000CVKN | Whole genome/VP1 phylogenetic tree | Fuzhou | 2018 |
| 2018FJPTN034 |  | NMDCN000CVRC | VP1 phylogenetic tree | Fuzhou | 2018 |
| 2018FJQZ003 |  | NMDCN000CVRD | VP1 phylogenetic tree | Quanzhou | 2018 |
| 2018FJQZ401 |  | NMDCN000CVRE | VP1 phylogenetic tree | Quanzhou | 2018 |
| 2018FJXM183 |  | NMDCN000CVRF | VP1 phylogenetic tree | Xiamen | 2018 |
| 2018FJXM580 |  | NMDCN000CVRG | VP1 phylogenetic tree | Xiamen | 2018 |
| 2019FJFZ072 |  | NMDCN000CVQN | VP1 phylogenetic tree | Fuzhou | 2019 |
| 2019FJFZ110 |  | NMDCN000CVKL | Whole genome/VP1 phylogenetic tree | Fuzhou | 2019 |
| 2019FJQZ053 |  | NMDCN000CVKK | Whole genome/VP1 phylogenetic tree | Quanzhou | 2019 |
| 2019FJQZ284 |  | NMDCN000CVQO | VP1 phylogenetic tree | Quanzhou | 2019 |
| 2019FJSM086 |  | NMDCN000CVQP | VP1 phylogenetic tree | Sanming | 2019 |
| 2019FJXM152 |  | NMDCN000CVQQ | VP1 phylogenetic tree | Xiamen | 2019 |
| 2019FJFZ150 |  | NMDCN000CVRH | VP1 phylogenetic tree | Fuzhou | 2019 |
| 2019FJLY213 |  | NMDCN000CVRI | VP1 phylogenetic tree | Longyan | 2019 |
| 2019FJND172 |  | NMDCN000CVRJ | VP1 phylogenetic tree | Ningde | 2019 |
| 2019FJNP084 |  | NMDCN000CVRK | VP1 phylogenetic tree | Nanping | 2019 |
| 2019FJNP107 |  | NMDCN000CVRL | VP1 phylogenetic tree | Nanping | 2019 |
| 2019FJNP228 |  | NMDCN000CVLE | Whole genome/VP1 phylogenetic tree | Nanping | 2019 |
| 2019FJQZ026 |  | NMDCN000CVRM | VP1 phylogenetic tree | Quanzhou | 2019 |
| 2019FJQZ175 |  | NMDCN000CVKJ | Whole genome/VP1 phylogenetic tree | Quanzhou | 2019 |
| 2019FJZZ077 |  | NMDCN000CVRN | VP1 phylogenetic tree | Zhangzhou | 2019 |
| 2019FJZZ358 |  | NMDCN000CVRO | VP1 phylogenetic tree | Zhangzhou | 2019 |
| 2020FJND007 |  | NMDCN000CVQR | VP1 phylogenetic tree | Ningde | 2020 |
| 2020FJSM038 |  | NMDCN000CVQS | VP1 phylogenetic tree | Sanming | 2020 |
| 2020FJSM043 |  | NMDCN000CVQT | VP1 phylogenetic tree | Sanming | 2020 |
| 2020FJSM081 |  | NMDCN000CVKH | Whole genome/VP1 phylogenetic tree | Sanming | 2020 |
| 2020FJXM101 |  | NMDCN000CVQU | VP1 phylogenetic tree | Xiamen | 2020 |
| 2020FJLY187 |  | NMDCN000CVKI | Whole genome/VP1 phylogenetic tree | Longyan | 2020 |
| 2020FJQZ058 |  | NMDCN000CVRP | VP1 phylogenetic tree | Quanzhou | 2020 |
| 2020FJSM049 |  | NMDCN000CVRQ | VP1 phylogenetic tree | Sanming | 2020 |
| 2020FJXM053 |  | NMDCN000CVRR | VP1 phylogenetic tree | Xiamen | 2020 |
| 2020FJXM196 |  | NMDCN000CVRS | VP1 phylogenetic tree | Xiamen | 2020 |
| 2021FJFZ246 |  | NMDCN000CVKG | Whole genome/VP1 phylogenetic tree | Fuzhou | 2021 |
| 2021FJQZ772 |  | NMDCN000CVQV | VP1 phylogenetic tree | Quanzhou | 2021 |
| 2021FJSM113 |  | NMDCN000CVR0 | VP1 phylogenetic tree | Sanming | 2021 |
| 2021FJFZ161 |  | NMDCN000CVRT | VP1 phylogenetic tree | Fuzhou | 2021 |
| 2021FJFZ571 |  | NMDCN000CVKF | Whole genome/VP1 phylogenetic tree | Fuzhou | 2021 |
| 2021FJLY071 |  | NMDCN000CVRU | VP1 phylogenetic tree | Longyan | 2021 |
| 2021FJLY232 |  | NMDCN000CVRV | VP1 phylogenetic tree | Longyan | 2021 |
| 2021FJLY602 |  | NMDCN000CVS0 | VP1 phylogenetic tree | Longyan | 2021 |
| 2021FJND173 |  | NMDCN000CVKE | Whole genome/VP1 phylogenetic tree | Ningde | 2021 |
| 2021FJNP317 |  | NMDCN000CVKD | Whole genome/VP1 phylogenetic tree | Nanping | 2021 |
| 2021FJNP584 |  | NMDCN000CVS1 | VP1 phylogenetic tree | Nanping | 2021 |
| 2021FJQZ715 |  | NMDCN000CVS2 | VP1 phylogenetic tree | Quanzhou | 2021 |
| 2021FJSM225 |  | NMDCN000CVS3 | VP1 phylogenetic tree | Sanming | 2021 |
| 2021FJSM298 |  | NMDCN000CVS4 | VP1 phylogenetic tree | Sanming | 2021 |
| 2021FJXM330 |  | NMDCN000CVS5 | VP1 phylogenetic tree | Xiamen | 2021 |
| 2021FJXM453 |  | NMDCN000CVKC | Whole genome/VP1 phylogenetic tree | Xiamen | 2021 |
| 2022FJFZ261 |  | NMDCN000CVS6 | VP1 phylogenetic tree | Fuzhou | 2022 |
| 2022FJFZ309 |  | NMDCN000CVS7 | VP1 phylogenetic tree | Fuzhou | 2022 |
| 2022FJLY187 |  | NMDCN000CVS8 | VP1 phylogenetic tree | Longyan | 2022 |
| 2022FJPT022 |  | NMDCN000CVS9 | VP1 phylogenetic tree | Putian | 2022 |
| 2022FJPT158 |  | NMDCN000CVSA | VP1 phylogenetic tree | Putian | 2022 |
| 2022FJQZ063 |  | NMDCN000CVSB | VP1 phylogenetic tree | Quanzhou | 2022 |
| 2022FJQZ106 |  | NMDCN000CVSC | VP1 phylogenetic tree | Quanzhou | 2022 |
| 2022FJSM023 |  | NMDCN000CVSD | VP1 phylogenetic tree | Sanming | 2022 |
| 2022FJSM054 |  | NMDCN000CVSE | VP1 phylogenetic tree | Sanming | 2022 |
| 2022FJSM249 |  | NMDCN000CVSF | VP1 phylogenetic tree | Sanming | 2022 |
| 2022FJXM013 |  | NMDCN000CVSG | VP1 phylogenetic tree | Xiamen | 2022 |
| 2022FJXM032 |  | NMDCN000CVKB | Whole genome/VP1 phylogenetic tree | Xiamen | 2022 |
| 2022FJXM049 |  | NMDCN000CVSH | VP1 phylogenetic tree | Xiamen | 2022 |
| 2022FJXM084 |  | NMDCN000CVSI | VP1 phylogenetic tree | Xiamen | 2022 |
| 2022FJXM143 |  | NMDCN000CVSJ | VP1 phylogenetic tree | Xiamen | 2022 |
| 2022FJXM209 |  | NMDCN000CVSK | VP1 phylogenetic tree | Xiamen | 2022 |
| 2022FJZZ202 |  | NMDCN000CVSL | VP1 phylogenetic tree | Zhangzhou | 2022 |
| 2023FJNDGT046 |  | NMDCN000CVLF | VP1 phylogenetic tree | Ningde | 2023 |
| 2023FJNDPN017 |  | NMDCN000CVKA | Whole genome/VP1 phylogenetic tree | Ningde | 2023 |
| 2023FJNPGZ029 |  | NMDCN000CVLG | VP1 phylogenetic tree | Nanping | 2023 |
| 2023FJNPGZ038 |  | NMDCN000CVLH | VP1 phylogenetic tree | Nanping | 2023 |
| 2023FJQZDY014 |  | NMDCN000CVLI | VP1 phylogenetic tree | Quanzhou | 2023 |
| 2023FJZZZA116 |  | NMDCN000CVLJ | VP1 phylogenetic tree | Zhangzhou | 2023 |
| 2023FJFZ149 |  | NMDCN000CVR1 | VP1 phylogenetic tree | Fuzhou | 2023 |
| 2023FJZZYX091 |  | NMDCN000CVR2 | VP1 phylogenetic tree | Zhangzhou | 2023 |
| 2023FJLY122 |  | NMDCN000CVSM | VP1 phylogenetic tree | Longyan | 2023 |
| 2023FJPTHJ001 |  | NMDCN000CVSN | VP1 phylogenetic tree | Putian | 2023 |
| 2023FJQZ091 |  | NMDCN000CVSO | VP1 phylogenetic tree | Quanzhou | 2023 |
| 2023FJQZNA040 |  | NMDCN000CVSP | VP1 phylogenetic tree | Quanzhou | 2023 |
| 2023FJQZSS033 |  | NMDCN000CVSQ | VP1 phylogenetic tree | Quanzhou | 2023 |
| 2023FJSM006 |  | NMDCN000CVSR | VP1 phylogenetic tree | Sanming | 2023 |
| 2023FJSMQL011 |  | NMDCN000CVSS | VP1 phylogenetic tree | Sanming | 2023 |
| 2023FJSMSX024 |  | NMDCN000CVST | VP1 phylogenetic tree | Sanming | 2023 |
| 2023FJZZ330 |  | NMDCN000CVK9 | Whole genome/VP1 phylogenetic tree | Zhangzhou | 2023 |
| 2024FJNDJC004 |  | NMDCN000CVK8 | Whole genome/VP1 phylogenetic tree | Ningde | 2024 |
| 2024FJNDJC12 |  | NMDCN000CVLD | VP1 phylogenetic tree | Ningde | 2024 |
| 2024FJNDJC015 |  | NMDCN000CVLL | VP1 phylogenetic tree | Ningde | 2024 |
| 2024FJNDZN038 |  | NMDCN000CVLM | VP1 phylogenetic tree | Ningde | 2024 |
| 2024FJPTN08 |  | NMDCN000CVLN | VP1 phylogenetic tree | Fuzhou | 2024 |
| 2024FJPTN097 |  | NMDCN000CVLO | VP1 phylogenetic tree | Fuzhou | 2024 |
| 2024FJPTN113 |  | NMDCN000CVK5 | Whole genome/VP1 phylogenetic tree | Fuzhou | 2024 |
| 2024FJQZDH063 |  | NMDCN000CVLP | VP1 phylogenetic tree | Quanzhou | 2024 |
| 2024FJQZQG091 |  | NMDCN000CVK4 | Whole genome/VP1 phylogenetic tree | Quanzhou | 2024 |
| 2024FJXM06 |  | NMDCN000CVLQ | VP1 phylogenetic tree | Xiamen | 2024 |
| 2024FJXMHL041 |  | NMDCN000CVK3 | Whole genome/VP1 phylogenetic tree | Xiamen | 2024 |
| 2024FJXMHL069 |  | NMDCN000CVLR | VP1 phylogenetic tree | Xiamen | 2024 |
| 2024FJXMSM158 |  | NMDCN000CVK2 | Whole genome/VP1 phylogenetic tree | Xiamen | 2024 |
| 2024FJXMSM203 |  | NMDCN000CVLS | VP1 phylogenetic tree | Xiamen | 2024 |
| 2024FJXMSM258 |  | NMDCN000CVLT | VP1 phylogenetic tree | Xiamen | 2024 |
| 2024FJXMTA135 |  | NMDCN000CVLU | VP1 phylogenetic tree | Xiamen | 2024 |
| 2024FJZZ116 |  | NMDCN000CVLV | VP1 phylogenetic tree | Zhangzhou | 2024 |
| 2024FJZZ120 |  | NMDCN000CVK1 | Whole genome/VP1 phylogenetic tree | Zhangzhou | 2024 |
| 2024FJZZ172 |  | NMDCN000CVM0 | VP1 phylogenetic tree | Zhangzhou | 2024 |
| 2024FJZZCT009 |  | NMDCN000CVK0 | Whole genome/VP1 phylogenetic tree | Zhangzhou | 2024 |
| 2024FJZZPH035 |  | NMDCN000CVM1 | VP1 phylogenetic tree | Zhangzhou | 2024 |
| 2024FJZZXC127 |  | NMDCN000CVM2 | VP1 phylogenetic tree | Zhangzhou | 2024 |
| 2024FJZZYX046 |  | NMDCN000CVM3 | VP1 phylogenetic tree | Zhangzhou | 2024 |
| 2024FJZZZA06 |  | NMDCN000CVJV | Whole genome/VP1 phylogenetic tree | Zhangzhou | 2024 |
| 2024FJFZ103 |  | NMDCN000CVSU | VP1 phylogenetic tree | Fuzhou | 2024 |
| 2024FJNPPC028 |  | NMDCN000CVK7 | Whole genome/VP1 phylogenetic tree | Nanping | 2024 |
| 2024FJPTCX113 |  | NMDCN000CVK6 | Whole genome/VP1 phylogenetic tree | Putian | 2024 |
| 2024FJXMJM005 |  | NMDCN000CVSV | VP1 phylogenetic tree | Xiamen | 2024 |
| 2024FJXMXA013 |  | NMDCN000CVT0 | VP1 phylogenetic tree | Xiamen | 2024 |
| 2024FJZZXC092 |  | NMDCN000CVT1 | VP1 phylogenetic tree | Zhangzhou | 2024 |
| 2024FJFZ158 |  | NMDCN000CVLK | VP1 phylogenetic tree | Fuzhou | 2025 |
| 2025FJFZ021 |  | NMDCN000CVM4 | VP1 phylogenetic tree | Fuzhou | 2025 |
| 2025FJFZ088 |  | NMDCN000CVM5 | VP1 phylogenetic tree | Fuzhou | 2025 |
| 2025FJNDJC018 |  | NMDCN000CVM6 | VP1 phylogenetic tree | Ningde | 2025 |
| 2025FJNPWYS071 |  | NMDCN000CVM7 | VP1 phylogenetic tree | Nanping | 2025 |
| 2025FJNPZH033 |  | NMDCN000CVJT | Whole genome/VP1 phylogenetic tree | Nanping | 2025 |
| 2025FJPTCX001 |  | NMDCN000CVM8 | VP1 phylogenetic tree | Putian | 2025 |
| 2025FJPTCX032 |  | NMDCN000CVM9 | VP1 phylogenetic tree | Putian | 2025 |
| 2025FJPTN007 |  | NMDCN000CVMA | VP1 phylogenetic tree | Fuzhou | 2025 |
| 2025FJQZSS006 |  | NMDCN000CVJS | Whole genome/VP1 phylogenetic tree | Quanzhou | 2025 |
| 2025FJQZSS032 |  | NMDCN000CVMB | VP1 phylogenetic tree | Quanzhou | 2025 |
| 2025FJSMDT001 |  | NMDCN000CVMC | VP1 phylogenetic tree | Sanming | 2025 |
| 2025FJSMQL005 |  | NMDCN000CVMD | VP1 phylogenetic tree | Sanming | 2025 |
| 2025FJSMSY053 |  | NMDCN000CVME | VP1 phylogenetic tree | Sanming | 2025 |
| 2025FJXMHL001 |  | NMDCN000CVMF | VP1 phylogenetic tree | Xiamen | 2025 |
| 2025FJXMSM011 |  | NMDCN000CVMG | VP1 phylogenetic tree | Xiamen | 2025 |
| 2025FJXMTA012 |  | NMDCN000CVMH | VP1 phylogenetic tree | Xiamen | 2025 |
| 2025FJZZ026 |  | NMDCN000CVMI | VP1 phylogenetic tree | Zhangzhou | 2025 |
| 2025FJZZLW003 |  | NMDCN000CVMJ | VP1 phylogenetic tree | Zhangzhou | 2025 |
| 2025FJZZNJ004 |  | NMDCN000CVMK | VP1 phylogenetic tree | Zhangzhou | 2025 |
| 2025FJZZNJ029 |  | NMDCN000CVML | VP1 phylogenetic tree | Zhangzhou | 2025 |
| F253501020053 |  | NMDCN000CVMM | VP1 phylogenetic tree | Fuzhou | 2025 |
| F253501020138 |  | NMDCN000CVMN | VP1 phylogenetic tree | Fuzhou | 2025 |
| F253501020229 |  | NMDCN000CVMO | VP1 phylogenetic tree | Fuzhou | 2025 |
| F253501020302 |  | NMDCN000CVMP | VP1 phylogenetic tree | Fuzhou | 2025 |
| F2535020059 |  | NMDCN000CVMQ | VP1 phylogenetic tree | Xiamen | 2025 |
| F253502010139 |  | NMDCN000CVMR | VP1 phylogenetic tree | Xiamen | 2025 |
| F253502010416 |  | NMDCN000CVJO | Whole genome/VP1 phylogenetic tree | Xiamen | 2025 |
| F253502010700 |  | NMDCN000CVMS | VP1 phylogenetic tree | Xiamen | 2025 |
| F25350201616 |  | NMDCN000CVMT | VP1 phylogenetic tree | Xiamen | 2025 |
| F253503040059 |  | NMDCN000CVMU | VP1 phylogenetic tree | Putian | 2025 |
| F253503040067 |  | NMDCN000CVMV | VP1 phylogenetic tree | Putian | 2025 |
| F253503040097 |  | NMDCN000CVJP | Whole genome/VP1 phylogenetic tree | Putian | 2025 |
| F253503040161 |  | NMDCN000CVN0 | VP1 phylogenetic tree | Putian | 2025 |
| F253503040200 |  | NMDCN000CVN1 | VP1 phylogenetic tree | Putian | 2025 |
| F253504040002 |  | NMDCN000CVN2 | VP1 phylogenetic tree | Sanming | 2025 |
| F253504040007 |  | NMDCN000CVN3 | VP1 phylogenetic tree | Sanming | 2025 |
| F253504040014 |  | NMDCN000CVN4 | VP1 phylogenetic tree | Sanming | 2025 |
| F253504040065 |  | NMDCN000CVN5 | VP1 phylogenetic tree | Sanming | 2025 |
| F253504040158 |  | NMDCN000CVN6 | VP1 phylogenetic tree | Sanming | 2025 |
| F253505030002 |  | NMDCN000CVN7 | VP1 phylogenetic tree | Quanzhou | 2025 |
| F253505030111 |  | NMDCN000CVN8 | VP1 phylogenetic tree | Quanzhou | 2025 |
| F253505030228 |  | NMDCN000CVN9 | VP1 phylogenetic tree | Quanzhou | 2025 |
| F253505030251 |  | NMDCN000CVNA | VP1 phylogenetic tree | Quanzhou | 2025 |
| F253505030284 |  | NMDCN000CVNB | VP1 phylogenetic tree | Quanzhou | 2025 |
| F253506030126 |  | NMDCN000CVNC | VP1 phylogenetic tree | Zhangzhou | 2025 |
| F253506030172 |  | NMDCN000CVND | VP1 phylogenetic tree | Zhangzhou | 2025 |
| F253506030199 |  | NMDCN000CVNE | VP1 phylogenetic tree | Zhangzhou | 2025 |
| F253506030226 |  | NMDCN000CVNF | VP1 phylogenetic tree | Zhangzhou | 2025 |
| F253506030258 |  | NMDCN000CVNG | VP1 phylogenetic tree | Zhangzhou | 2025 |
| F2535070200256 |  | NMDCN000CVJR | Whole genome/VP1 phylogenetic tree | Nanping | 2025 |
| F253507020066 |  | NMDCN000CVNH | VP1 phylogenetic tree | Nanping | 2025 |
| F253507020094 |  | NMDCN000CVNI | VP1 phylogenetic tree | Nanping | 2025 |
| F253507020141 |  | NMDCN000CVNJ | VP1 phylogenetic tree | Nanping | 2025 |
| F253507020227 |  | NMDCN000CVNK | VP1 phylogenetic tree | Nanping | 2025 |
| F253508020028 |  | NMDCN000CVJU | Whole genome/VP1 phylogenetic tree | Longyan | 2025 |
| F253508020110 |  | NMDCN000CVNL | VP1 phylogenetic tree | Longyan | 2025 |
| F253508020257 |  | NMDCN000CVNM | VP1 phylogenetic tree | Longyan | 2025 |
| F253508020267 |  | NMDCN000CVNN | VP1 phylogenetic tree | Longyan | 2025 |
| F253508020283 |  | NMDCN000CVNO | VP1 phylogenetic tree | Longyan | 2025 |
| F253509020029 |  | NMDCN000CVNP | VP1 phylogenetic tree | Ningde | 2025 |
| F253509020035 |  | NMDCN000CVJQ | Whole genome/VP1 phylogenetic tree | Ningde | 2025 |
| F253509020046 |  | NMDCN000CVNQ | VP1 phylogenetic tree | Ningde | 2025 |
| F253509020096 |  | NMDCN000CVNR | VP1 phylogenetic tree | Ningde | 2025 |
| F253509020116 |  | NMDCN000CVNS | VP1 phylogenetic tree | Ningde | 2025 |
| F253509020154 |  | NMDCN000CVNT | VP1 phylogenetic tree | Ningde | 2025 |
| 2025FJFZ083 |  | NMDCN000CVJK | Whole genome/VP1 phylogenetic tree | Fuzhou | 2025 |
| 2025FJNDJC007 |  | NMDCN000CVT2 | VP1 phylogenetic tree | Ningde | 2025 |
| 2025FJNDPN052 |  | NMDCN000CVT3 | VP1 phylogenetic tree | Ningde | 2025 |
| 2025FJNDPN081 |  | NMDCN000CVT4 | VP1 phylogenetic tree | Ningde | 2025 |
| 2025FJNPZH011 |  | NMDCN000CVJH | Whole genome/VP1 phylogenetic tree | Nanping | 2025 |
| 2025FJNPZH022 |  | NMDCN000CVT5 | VP1 phylogenetic tree | Nanping | 2025 |
| 2025FJQZSS004 |  | NMDCN000CVJM | Whole genome/VP1 phylogenetic tree | Quanzhou | 2025 |
| 2025FJQZTS053 |  | NMDCN000CVT6 | VP1 phylogenetic tree | Quanzhou | 2025 |
| 2025FJXMJM009 |  | NMDCN000CVT7 | VP1 phylogenetic tree | Xiamen | 2025 |
| 2025FJXMSM003 |  | NMDCN000CVJG | Whole genome/VP1 phylogenetic tree | Xiamen | 2025 |
| F253503040055 |  | NMDCN000CVJJ | Whole genome/VP1 phylogenetic tree | Putian | 2025 |
| F253508020002 |  | NMDCN000CVJI | Whole genome/VP1 phylogenetic tree | Longyan | 2025 |
| 2025FJNDSN001 |  | NMDCN000CVJL | Whole genome phylogenetic tree | Ningde | 2025 |
| F253504040207 |  | NMDCN000CVJN | Whole genome phylogenetic tree | Sanming | 2025 |

**Supplementary Figure 1.** Distribution of whole-genome (WGS) and VP1 sequences by city in Fujian Province.

**Note:** The base map is based on the standard map (No. GS(2024)0650) provided by the National Platform for Common Geospatial Information Services (https://www.tianditu.gov.cn/). The original boundary of the map has not been modified. The bar chart displays the counts of WGS (purple) versus VP1 (light green) sequences obtained from each city.

**Supplementary Figure 2.** The Age Distribution of CVA16-associated HFMD case in Fujian Province from 2010 to 2025. This figure provides a detailed breakdown of the ≥7 years age group (originally aggregated in Figure 3B) into finer strata (7, 8, 9, and ≥10 years), revealing distinct fluctuation patterns among older populations.
